# Expression profiling of clonal lymphocyte cell cultures from Rett syndrome patients

**DOI:** 10.1186/1471-2350-7-61

**Published:** 2006-07-21

**Authors:** Ivan J Delgado, Dong Sun Kim, Karen N Thatcher, Janine M LaSalle, Ignatia B Van den Veyver

**Affiliations:** 1 Department of Obstetrics and Gynecology, Baylor College of Medicine, Houston, TX, USA; 2 Medical Microbiology and Immunology and Rowe Program in Human Genetics, School of Medicine, University of California, Davis, CA, USA; 3 Department of Molecular and Human Genetics, Baylor College of Medicine, Houston, TX, USA; 4 Senior Scientist, Identigene Inc., 5615 Kirby, Suite 800 Houston, TX 77005, USA; 5 Assistant Professor, Department of Anatomy, School of Medicine, Kyungpook National University, South Korea

## Abstract

**Background:**

More than 85% of Rett syndrome (RTT) patients have heterozygous mutations in the X-linked *MECP2 *gene which encodes methyl-CpG-binding protein 2, a transcriptional repressor that binds methylated CpG sites. Because *MECP2 *is subject to X chromosome inactivation (XCI), girls with RTT express either the wild type or mutant *MECP2 *in each of their cells. To test the hypothesis that *MECP2 *mutations result in genome-wide transcriptional deregulation and identify its target genes in a system that circumvents the functional mosaicism resulting from XCI, we performed gene expression profiling of pure populations of untransformed T-lymphocytes that express either a mutant or a wild-type allele.

**Methods:**

Single T lymphocytes from a patient with a c.473C>T (p.T158M) mutation and one with a c.1308-1309delTC mutation were subcloned and subjected to short term culture. Gene expression profiles of wild-type and mutant clones were compared by oligonucleotide expression microarray analysis.

**Results:**

Expression profiling yielded 44 upregulated genes and 77 downregulated genes. We compared this gene list with expression profiles of independent microarray experiments in cells and tissues of RTT patients and mouse models with *Mecp2 *mutations. These comparisons identified a candidate MeCP2 target gene, *SPOCK1*, downregulated in two independent microarray experiments, but its expression was not altered by quantitative RT-PCR analysis on brain tissues from a RTT mouse model.

**Conclusion:**

Initial expression profiling from T-cell clones of RTT patients identified a list of potential MeCP2 target genes. Further detailed analysis and comparison to independent microarray experiments did not confirm significantly altered expression of most candidate genes. These results are consistent with other reported data.

## Background

Rett syndrome (RTT, OMIM 312750) is an X-linked neurodevelopmental disorder that affects 1 in 10,000 to 15,000 females [1,2]. Girls with RTT have an apparently normal early development, followed by deceleration of head growth, loss of language skills, loss of purposeful hand movements and impaired social contact. As the disease progresses they develop respiratory abnormalities, autistic features, stereotypic hand movements, scoliosis, general growth delay, seizures and ataxia [3,4]. RTT is caused by heterozygous mutations in the methyl-CpG-binding protein 2 gene (*MECP2*), an X-linked gene subject to X chromosome inactivation (XCI) [5]. Mutations in the coding region of this gene are detected in 85% of patients with classic RTT [6-9]. An additional 10% have large deletions affecting several exons of *MECP2 *[10-12]. Alternative splice variants of *MECP2 *have been identified [9,13,14] that result in two protein isoforms. MeCP2-e1 (MeCP2α/B) is encoded by exons 1, 3 and 4 and is more abundant in brain than the previously identified MeCP2-e2 (MeCP2β/A) isoform, which is encoded by exons 2, 3 and 4. Interestingly, mutations in exon 1 are only rarely found in RTT patients [9,15,16]. Both isoforms of MeCP2 are identical beyond exon 2 and contain an 84-amino acid methyl-CpG-binding domain [17] and a 104-amino acid transcriptional repression domain (TRD) [18] as well as a C-terminal protein interaction domain. MeCP2 has been shown to bind DNA, preferentially at methylated CpG dinucleotides with resulting transcriptional repression of nearby genes through the recruitment of a histone deacetylase (HDAC1 and 2) and a Sin3A-containing corepressor complex [19,20]. MeCP2 also associates with histone methyltransferase activity and the DNA methyltransferase DNMT1 [21,22]. Brahma (Brm), the catalytic component of the SWI/SNF ATPase-dependent remodelling complex, was found to interact with MeCP2 [23], extending the mechanistic link between DNA methylation, chromatin remodelling and transcriptional repression. Recently, MecP2 has also been demonstrated to regulate alternative splicing and interact with an RNA-binding protein (Y box-binding protein 1) [24].

Despite active research since the discovery of *MECP2 *mutations in RTT, it has proven difficult to identify other direct target genes for the proposed functions of MeCP2. Candidate gene-based approaches using vertebrate models with disrupted MeCP2 have resulted in the identification of brain-derived neurotrophic factor (*Bdnf*) [25-27] and *Hairy2a *[28] as MeCP2 targets. MeCP2 binds to methylated CpG sites near promoter III of *BDNF *in resting neurons [25,26], and disease progression in a RTT mouse model correlates inversely with Bdnf expression [27]. Hairy2a is upregulated in the absence of MeCP2 in *Xenopus *embryos [28]. Following the hypothesis that MeCP2 functions primarily as a transcriptional repressor, several groups have attempted to screen for its targets by transcriptional profiling using RNA from postmortem brain tissues or cell lines derived from RTT patients, or from tissues of mice with engineered mutations in *Mecp2*. In one study, 70 transcripts were found to have altered gene expression in mutant versus wild-type fibroblast clones and lymphoblastoid cells lines [29]. The authors concluded that MeCP2 deficiency did not lead to global deregulation of gene expression and suggested that clonal fibroblast lines may show substantial variation, making them an unstable resource for expression profiling studies. In addition, lymphoblastoid cell lines are immortalized by Epstein-Barr virus (EBV) transformation, which can alter their transcriptional profile and methylation status. Expression profiling of brain from male mice with a deletion of *Mecp2 *also yielded only few genes with altered expression between wild-type and mutant mice [30]. Upon further analysis, those identified fell well within the range of the high false-positive rate [30]. In a third transcriptional profiling microarray study on postmortem RTT brains, significant changes in expression for 135 genes on three different cDNA microarrays were found [31]. Yet, the small sample size analyzed and the validation of the array data with the same samples instead of independent samples may have led to false-positive results [30]. It has been hypothesized that transcriptional profiling of *MECP2*-deficient brains has failed to identify MeCP2-regulated genes because of the high complexity in regional organization and the admixture of neuronal and non-neuronal cell types that is inherent to brain tissue [32]. Perhaps MeCP2 targets are also difficult to identify by microarray analysis because the absolute changes in expression level for individual genes are very small [33]. A more direct approach that circumvents this problem is the use of a chromatin immunoprecipitation (ChIP) strategy to directly search for the sites of MeCP2-binding to DNA. This led to the recent identification of the *DLX5 *gene as a MeCP2 target and other genes such as *CNTN2*, *FOXA3*, and *SIAT4A *as candidate genes regulated by MeCP2 [34].

In the present study we sought to overcome some of the limitations of transcriptional profiling on complex tissues, such as brain, as well as those of transformed lymphoblastoid cell lines by studying clonal cultures of non-transformed lymphocytes from individual RTT patients. These cells can be easily obtained through blood sampling, readily cultured and subjected to single-cell subcloning. Because *MECP2 *undergoes XCI, this last feature allows the separation of cells that express the wild-type *MECP2 *from the active X from those cells that express the mutant *MECP2 *from the active X. To identify downstream targets of MeCP2, we compared global gene expression patterns in matched pairs of clonally derived mutant or wild-type *MECP2*-expressing lymphocyte cultures from two girls with classic Rett syndrome. Our microarray analysis revealed 121 genes with changes in expression between cells expressing the wild-type *MECP2 *and those expressing the mutant *MECP2*. We compared this gene list to those of other transcriptional profiling experiments and focused further analysis on a downregulated putative MeCP2 target, *SPOCK1*. However, quantitative RT-PCR analysis on RNA from the clonal T-lymphocytes, differentiating mouse embryoid bodies (EB) and various mouse brain regions did not reveal significantly different expression between mutant and wild-type RNAs.

## Methods

### T-lymphocyte single cell cloning

Peripheral blood mononuclear cells (PBMCs) were isolated by Ficoll-Hypaque from fresh blood samples of Rett syndrome patients, collected under a research protocol approved by the Baylor College of Medicine Institutional Review Board for Human Subjects Research. Isolation of T-lymphocyte clones has been described previously [35-37]. Briefly, PBMCs from the patients were plated at limiting dilution in 96-well plates in the presence of irradiated allogeneic PBMCs in RPMI media containing 20% pooled human sera (Sigma-Aldrich, St. Louis, MO), 2 μg/mL PHA.P (Murex diagnostics, Inc. Dartford Kent, UK) and 5% Human T-stim (Collaborative Biomedical Products, Bedford, MA). Nine to ten days following original culture, wells showing clonal positive growth were restimulated and cultured for an additional 7–14 days. Additional restimulations were performed every 10–12 days until >10^7 ^cells were obtained from each clone.

### RNA isolation and reverse transcription

Total RNA was isolated from T-cell clones using Trizol reagent (Invitrogen Corp., Carlsbad, CA). RNA was treated with 1 U of RNase-free DNase I (Ambion Inc., Austin, TX). RNA samples were stored at -80°C until later use. Analysis of allelic expression of *MECP2 *by RT-PCR and restriction digestion to verify clonality of expanded lymphocyte clones from patients RT208 (c.1308-1309delTC mutation) and RT211 (p.T158M mutation) has been previously described [37].

Animal research was performed under a protocol approved by the Baylor College of Medicine animal research review board. After euthanasia, brain regions (cortex, cerebellum, olfactory bulb) were quickly removed from 5 week-old mice and immediately frozen in liquid nitrogen. E16.5 embryos were collected and heads and bodies immediately frozen in liquid nitrogen. Total RNA was extracted using the Qiagen RNeasy Mini kit (Qiagen Inc., Valencia, CA). Prior to array hybridization, the RNA quality and degradation was verified using the Agilent 2100 Bioanalyzer (Agilent Technologies, Palo Alto, CA). In addition, quality control parameters were assessed throughout the experimental process to assure the efficiency of transcription, integrity of hybridization, and consistency of qualitative calls. Assessment during the synthesis of the hybridization transcript was accomplished by spectrophotometric analysis of the starting RNA and cRNA, and by gel electrophoresis following synthesis of the cDNA, cRNA and fragmentation of the cRNA. A 3'/5' ratio of *GAPDH *less than 2 was considered acceptable for efficiency of transcription. Spiked control transcripts were also monitored to verify hybridization integrity. Scaling factors for each sample ranged between 1.72 and 5.39. All quality control measures were consistent with the manufacturers recommended procedures and conformed to the recommended cut-off values except for one of the RT211 (p.T158M) wild-type clonal lymphocyte samples (Wt5) which was not included in subsequent analysis because the RNA was found to be partially degraded.

### Oligonucleotide expression array hybridization

Total RNA was converted to double-stranded cDNA (ds-cDNA) in the presence of an oligo-dT primer containing a T7 RNA polymerase promoter. *In vitro *transcription was performed to generate biotin-labeled cRNA from the ds-cDNA template in the presence of a mixture of biotin-labeled ribonucleotides. Biotin-labeled cRNA (15 μg) was fragmented to a size range between 50–200 bases in length and used to hybridize to Affymetrix human HG-U133A chips. After hybridization, the arrays were washed and stained with Streptavidin-phycoerythrin. Arrays were read at a resolution of 3 microns using a HP Gene Array Scanner (Hewlett Packard, Inc., Palo Alto CA) The average background for each chip was determined to be below the common threshold of 100 for all analyzed chips.

### Microarray data analysis

**Figure 1:**
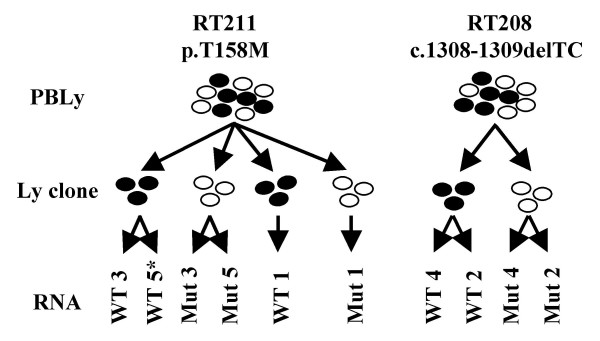
**Experimental design of T-lymphocyte clone isolation**. Two technical replicate hybridizations for the wild type *MECP2 *and mutant *MECP2*-expressing clonal cell-cultures (RT211 with p.T158M mutation and RT208 with c. 1308-1309delTC mutation) were performed, as well as a biological replicate hybridization with an independent clone of patient RT211. (PBLy = peripheral blood lymphocytes; Ly clone = T-lymphocyte clone; * indicates unused in array hybridization analysis because of insufficient RNA quality)

For the array hybridizations with RNA from clonal lymphocyte cultures, raw data was obtained from an HP Gene Array Scanner and normalized by the GC Robust Multichip Average or GC-RMA analysis. Briefly, in RMA analysis [38,39], described by Irazarry et al. [40,41], the background correction is based on the distribution of probe mismatch (MM) values amongst all probes on the array and compensates for non-specific binding using "perfect match" (PM) distributions rather than PM-MM values. The RMA method uses probe-level multichip quantile normalization to unify PM distributions across all chips and robust probe-set summary of the log-normalized probe-level data by median polishing. GC-RMA is similar to RMA, except that it reduces the bias of not substracting MM values in the background correction by an algorithm that takes into account that the non-specific affinity of a probe is related to its sequence [39,42]. GC-RMA normalizations were performed using GeneSpring GX 7.3.1 software (Agilent Technologies, Palo Alto, CA) with default parameters as follows: values below 0.01 were set to 0.01; each measurement was divided by the 50.0th percentile of all measurements in that sample; each gene was divided by the median of its measurements in all samples; if the median of the raw values was below 10 then each measurement for that gene was divided by 10 if the numerator was above 10, otherwise the measurement was thrown out. Log2-transformed data files from all GC-RMA normalizations were analyzed for significance using the GeneSpring GX 7.3.1 software with the most recent annotations of the HG-U133A human genome chip. Samples from 9 chips were divided in 4 groups according to genotype (wild-type versus mutant) and specific patient (RT211 with the p.T158M mutation versus RT208 with the c.1308-1309delTC). As outlined above, we excluded one wild-type chip (Wt5) from analysis because of poor RNA quality (Figure 1). Parametric testing (one-way ANOVA with a p-value cut-off of <0.05) was used; variances were calculated with the cross-gene error model. When multiple-testing correction was performed, the Benjamini-Hochberg false discovery rate (FDR) analysis was used. The lists were also filtered for two-fold and 1.5-fold up or down-regulation.

Gene lists of all up and downregulated genes at the p < 0.05 level were then compared to lists of genes from analysis of another transcriptional profiling experiment in progress in our laboratory that compares gene expression profiles of *Mecp2*^*R308/Y*^-mutant [43] and wild-type embryoid bodies (EB) at day 5 after retinoic acid-induced *in vitro *differentiation of mouse embryonic stem cells (unpublished data). We also compared the gene lists from the clonal lymphocyte cultures to those from previously published microarray experiments on RNA samples of Rett syndrome patients or of *Mecp2*-mutant mouse models [29-31,44], and genes found to be MeCP2-targets by chromatin immunoprecipitation experiments [34]. To allow direct comparison of all different datasets using consistent nomenclature, we replaced the gene annotations in all these lists with the official symbol for the human genes (or human orthologue of mouse genes) from the NCBI Entrez Gene browser [45] (See additional file 1: supplementary table 1).

### Quantitative real-time PCR

For quantitative real-time PCR on mouse tissues, an equal amount of total RNA isolated from brain regions (cortex, cerebellum and olfactory bulb) of 5 week-old mice or E16.5 embryos was converted to cDNA with SuperScript II reverse transcriptase (Invitrogen Corp., Carlsbad, CA). For quantitative real-time PCR on human samples, total RNA from the clonal lymphocyte cultures used for microarray hybridization was similarly converted to cDNA. We used the ABI Prism 7300 sequence detection system (Applied Biosystems, Foster City, CA) to perform quantitative real time PCR with SYBR Green as the detection agent. The primers used were as follows. Murine *Spock1 *(NM_009262): *Spock1 *forward primer: TGCACGGACAAGGAGCTGCG, *Spock1 *reverse primer: GAACCAGTCCTTCAGCCGG; Murine *Gapdh *(NM_001001303): *Gapdh *forward primer: CATGGCCTTCCGTGTTCCTA, *Gapdh *reverse primer: GCGGCACGTCAGATCCA; human *SPOCK1 *(NM_004598): *SPOCK1 *forward primer: TGCACAGACAAGGAGTTGCG, *SPOCK1 *reverse primer: AAACCAATCCTTCAGCCGG; human *GAPDH *(NM_002046): *GAPDH *forward primer: TGGGCTACACTGAGCACCAG, and *GAPDH *reverse primer: GGGTGTCGCTGTTGAAGTCA. After PCR amplification, a dissociation protocol was performed to determine the melting curve of the PCR product. Only reactions with melting curves indicative of a single amplification product were analyzed further. The identity and expected size of the single PCR product was also confirmed by agarose gel electrophoresis. Relative quantification of the abundance of each gene at every time point was performed by the comparative ΔΔC_T _method as described in the Applied Biosystem user bulletin #2 [46]. The values obtained by this method are a measure of the fold-change in expression of the gene of interest compared to the calibrator sample, all normalized to *Gapdh *(mouse samples) or *GAPDH *(human samples). The Student's t test was used to determine statistical significance of expression differences between average C_T _values of wild-type and mutant samples. A p-value < 0.05 was considered significant.

## Results

### Isolation of *MECP2*-mutant and *MECP2*-wild type T-lymphocyte clones

Because *MECP2 *is an X-linked gene that undergoes XCI in females, each cell expresses *MECP2 *exclusively from only one of the two X chromosomes (the active X), while the copy of the gene on the inactive X chromosome is silenced. In order to separate cells that express the wild type *MECP2 *from those that express the mutant *MECP2 *allele, we performed single-cell cloning of T-lymphocytes isolated from peripheral blood mononuclear cells of RTT patients [37]. To allow for comparison between two types of mutations, samples from two girls with classic RTT, one (RT211) with a p.T158M missense mutation and one (RT208) with a c.1308-1309delTC frameshift mutation, were used for this experiment. Following two rounds of mitogenic stimulation, all clonal cultures with greater than 10^7 ^cells were harvested for RNA isolation. As previously reported for these cell cultures, RT-PCR followed by digestion of the amplified products to completion with restriction enzymes specific for the mutant allele, demonstrated that we had obtained pure populations of clonal cells, expressing either a mutant or a wild-type *MECP2 *[37].

### Microarray analysis of matched lymphocyte clones

Two technical replicate hybridizations were performed for one of each clone type, as well as an additional biological replicate hybridization for the wild-type *MECP2 *and mutant *MECP2 *clones of RT211 (p.T158M) on HG-U133A human genome chips containing 22,283 genes (Affymetrix, Santa Clara, CA) (Figure 1). Signals of all hybridized chips were normalized using the GC-RMA method [42]. We then performed one-way ANOVA analysis to find genes with significantly altered expression in Mut2 and Mut4 from patient RT208 (c.1308-1309delTC), and Mut1, Mut3 and Mut5 from patient RT211 (p.T158M) compared to WT2 and WT4 from patient RT208, and WT1 and WT3 from patient RT211.

One-way ANOVA analysis, with a p < 0.05 yielded a total of 121 genes with significantly different expression between wild-type and mutant samples. Of these, 44 had a reduced level of expression (Table 1) and 77 had an increased level of expression (Table 2) in mutant compared to wild-type clones. When these lists were filtered for absolute fold-expression change, there were 28 genes with 1.5 fold higher expression in mutant compared to wild-type (13 genes with 2-fold higher expression) and 21 genes with 1.5 fold lower expression in mutant compared to wild-type (12 genes with 2-fold lower expression). It is however important to note that, although these expression changes are statistically significant at a cut-off of p < 0.05, 1,134 (5%) of the 22,283 genes present in the HG-U133A chip, would randomly be expected to show altered expression. In addition, FDR analysis using the Benjamini-Hochberg algorithm did not retain any genes with significantly altered expression. Because FDR analysis may be too stringent for genes with low-level expression, we next determined if any of the 121 genes found to have altered expression without the FDR correction might be more relevant to Rett syndrome. We compared our data to those of other published expression microarray experiments [29-31,44], to a published list of putative direct MeCP2 targets found by chromatin immunoprecipitation [34], and to results from another ongoing microarray experiments in our laboratory that compares murine EBs with a truncating *Mecp2 *mutation (*Mecp2*^*R308/Y*^) to wild-type EBs at day 5 after retinoic-acid induced *in vitro *differentiation of mouse ES cells (unpublished data). Very limited overlap was found between each of these lists (See additional file 2: Supplementary table 2). One downregulated gene, *BEXL1*, encoding brain expressed X-linked-like 1, a protein of unknown function of the BEX family of proteins [47], was also found downregulated in the expression profiling performed by Traynor et al. [29]. However, its expression was not altered in our microarray experiments on *Mecp2*^*R308/Y *^mutant samples compared to wild-type differentiating mouse EBs, and it was not prioritized for further analysis. In contrast, two genes, *LPIN1 *and *SPOCK1 *with significantly reduced expression in this microarray analysis also showed lower expression in *Mecp2*^*R308/Y *^mutant samples compared to wild-type in our microarray experiments on differentiating mouse EBs. *LPIN1 *encodes lipin, a protein important for adipocyte differentiation and function that plays a role in glucose and lipid homeostasis and has been associated with human lipodystrophy [48,49]. Based on this known function, we considered it a less likely candidate for an important role in the neurological phenotype of RTT. We focused on *SPOCK1*, the only gene found downregulated in our own two microarray experiments that has a function that is compatible with a possible role in the RTT phenotype. *SPOCK1 *encodes the 439-amino acid SPOCK1 protein (Sparc/osteonectin-like domains, CWCV and Kazal-like domains proteoglycan 1 precursor), also known as Testican [50]. It is a predominantly extracellular matrix chondroitin sulphate-linked proteoglycan that is related to protein families that have protease inhibitor function and are involved in cell adhesion, migration, and proliferation. Human *SPOCK1 *and mouse *Spock1 *share 95% identity. *SPOCK1 *is highly enriched in neurons and endothelial cells of the central nervous system, absent in quiescent astroglia, but upregulated in activated astroglia [51]. Mouse *Spock1 *is highly expressed at the start of neurogenesis during neuron migration and axonal outgrowth and subsequently in developing synaptic fields [52]. Spock1 has also been found to inhibit adhesion and neurite outgrowth of cultured Neuro2A cells [53]. In the adult, it localizes predominantly to the postsynaptic region of a subpopulation of pyramidal neurons in the CA3 region of the hippocampus [54]. These features suggested a possible role in motor or behavioural aspects of the RTT phenotype. Therefore, we focused on this gene for further analysis.

**Table 1:** Genes with lower expression in mutant samples on microarray analysis. This table contains a list of genes with lower expression in mutant T-lymphocyte clones by one-way ANOVA analysis (p < 0.05). * indicates >1.5-fold change in expression level and ** indicates >2-fold change in expression level.

**No.**	**Gene Name**	**P-value**	**Common name**	**Genbank No.**	**Description**
1**	201160_s_at	0.000593	CSDA	AL556190	cold shock domain protein A
2*	201417_at	0.00108	SOX4	AL136179	SRY (sex determining region-Y) box 4
3	202565_s_at	0.00385	SVIL	NM_003174	supervillin
4	204860_s_at	0.00453	BIRC1	AI817801	Transcribed seq with strong similarity BIR1
5	215333_x_at	0.00459	GSTM1	X08020	glutathione S-transferase M1
6*	210763_x_at	0.00546	NCR3	AF031137	natural cytotoxicity triggering receptor 3
7**	217979_at	0.00821	TM4SF13	NM_014399	transmembrane 4 superfamily member 13
8	208524_at	0.0117	GPR15	NM_005290	G protein-coupled receptor 15
9**	215440_s_at	0.012	BEXL1	AL523320	hypothetical protein FLJ10097
10	213193_x_at	0.0157	TRBC1	AL559122	T cell receptor beta chain BV20S1 BJ1-5 BC1 mRNA
11	220131_at	0.0174	FXYD7	NM_022006	FXYD domain containing ion transport regulator 7
12	204550_x_at	0.0175	GSTM1	NM_000561	glutathione S-transferase M1
13	212739_s_at	0.0186	NME4	AL523860	non-metastatic cells 4, protein expressed in
14*	220684_at	0.0187	TBX21	NM_013351	T-box 21
15	203030_s_at	0.0195	PTPRN2	AF007555	protein tyrosine phosphatase, receptor type N polypeptide 2
16	219693_at	0.0232	AGPAT4	NM_020133	1-acylglycerol-3-phosphate O-acyltransferase 4
17*	219654_at	0.0245	PTPLA	NM_014241	protein tyrosine phosphatase-like, member a
18	217764_s_at	0.0245	RAB31	AF183421	RAB31, member RAS oncogene family
19*	217671_at	0.0247	RFX3	BE466926	regulatory factor X, 3 (Influences HLA class II expression)
20**	204232_at	0.025	FCER1G	NM_004106	Fc fragment of IgE, high affinity receptor for γ-polypeptide
21*	1405_i_at	0.0298	CCL5	M21121	chemokine (C-C motif) ligand 5
22**	206170_at	0.0308	ADRB2	NM_000024	adrenergic, beta-2-, receptor, surface
23**	211583_x_at	0.0317	NCR3	AF031136	natural cytotoxicity triggering receptor 3
24	202279_at	0.0328	C14orf2	NM_004894	chromosome 14 open reading frame 2
25	214012_at	0.0333	ARTS-1	BE551138	type 1 TNF receptor shedding aminopeptidase regulator
26	204396_s_at	0.0346	GRK5	NM_005308	G protein-coupled receptor kinase 5
27**	211010_s_at	0.0349	NCR3	AF031138	natural cytotoxicity triggering receptor 3
28	208791_at	0.0351	CLU	M25915	clusterin
29**	219529_at	0.0383	CLIC3	NM_004669	chloride intracellular channel 3
30	217104_at	0.0385	LOC283687	AL109714	hypothetical protein LOC283687
31	213280_at	0.0397	GARNL4	AK000478	GTPase activating RANGAP domain-like 4
32	202234_s_at	0.04	SLC16A1	BF511091	solute carrier family 16, member 1
33**	215006_at	0.0404		AK023816	CDNA FLJ13754 fis, clone PLACE3000362
34	204883_s_at	0.0409	HUS1	AI968626	HUS1 checkpoint homolog (S. pombe)
35**	217963_s_at	0.0409	NGFRAP1	NM_014380	nerve growth factor receptor (TNFRSF16) ass. protein 1
36**	212070_at	0.0419	GPR56	AL554008	G protein-coupled receptor 56
37**	202363_at	0.042	SPOCK1	AF231124	sparc/osteonectin, cwcv and kazal-like domains proteoglycan (testican)
38*	206974_at	0.0427	CXCR6	NM_006564	chemokine (C-X-C motif) receptor 6
39	200965_s_at	0.0433	ABLIM1	NM_006720	actin binding LIM protein 1
40	219457_s_at	0.045	RIN3	NM_024832	Ras and Rab interactor 3
41**	213915_at	0.0452	NKG7	NM_005601	natural killer cell group 7 sequence
42	215411_s_at	0.0463	C6orf4	AL008730	
43*	212274_at	0.0484	LPIN1	AV705559	AV705559ADB Homo sapiens cDNA clone ADBAPE04 5'
44	219155_at	0.049	PITPNC1	NM_012417	phosphatidylinositol transfer protein, cytoplasmic 1

**Table 2:** Genes with higher expression in mutant samples on microarray analysis. This table contains a list of genes with higher expression in mutant T-lymphocyte clones by one-way ANOVA analysis (p < 0.05). * indicates >1.5-fold change in expression level and ** indicates >2-fold change in expression level.

**No.**	**Gene Name**	**P-value**	**Common name**	**Genbank No.**	**Description**
1	219759_at	6.66E-11	LRAP	NM_022350	leukocyte-derived arginine aminopeptidase
2	206148_at	0.000152	IL3RA	NM_002183	interleukin 3 receptor, alpha (low affinity)
3*	213655_at	0.000209	YWHAE	AA502643	tyrosine 3-monooxygenase/tryptophan 5-monooxygenase activation protein
4*	212952_at	0.000278	MNT	AA910371	calreticulin
5	218340_s_at	0.000346	FLJ10808	NM_018227	hypothetical protein FLJ10808
6*	200765_x_at	0.000507	CTNNA1	NM_001903	catenin (cadherin-associated protein), alpha 1, 102 kDa
7*	210732_s_at	0.000704	LGALS8	AF342816	lectin, galactoside-binding, soluble, 8 (galectin 8)
8	219974_x_at	0.00131	ECHDC1	NM_018479	enoyl Coenzyme A hydratase domain containing 1
9	202732_at	0.00142	PKIG	NM_007066	protein kinase (cAMP-dependent, catalytic) inhibitor γ
10	204418_x_at	0.00183	GSTM4	NM_000848	glutathione S-transferase M2 (muscle)
11	221754_s_at	0.00187	CORO1B	AI341234	coronin, actin binding protein, 1B
12*	219648_at	0.00266	FLJ10116	NM_018000	hypothetical protein FLJ10116
13*	212099_at	0.00528	RHOB	AI263909	ras homolog gene family, member B
14	213135_at	0.00579	TIAM1	U90902	T-cell lymphoma invasion and metastasis 1
15**	210844_x_at	0.00603	CTNNA1	D14705	catenin (cadherin-associated protein), alpha 1, 102 kDa
16	208743_s_at	0.00617	YWHAB	BC001359	tyrosine 3-monooxygenase/tryptophan 5-monooxygenase activation protein
17*	204201_s_at	0.00621	PTPN13	NM_006264	protein tyrosine phosphatase, non-receptor type 13
18*	222146_s_at	0.00628	TCF4	AK026674	transcription factor 4
19*	214130_s_at	0.00717	PDE4DIP	AI821791	phosphodiesterase 4D interacting protein (myomegalin)
20**	211893_x_at	0.00833	CD6	U66145	CD6 antigen
21**	210136_at	0.00975	MBP	AW070431	myelin basic protein
22**	209012_at	0.0106	TRIO	AV718192	triple functional domain (PTPRF interacting)
23	222173_s_at	0.0109	TBC1D2	AK026105	TBC1 domain family, member 2
24	218435_at	0.0112	DNAJD1	NM_013238	DnaJ (Hsp40) homolog, subfamily D, member 1
25	210354_at	0.0122	IFNG	M29383	Human mRNA for HuIFN-gamma interferon.
26	217744_s_at	0.0132	PERP	NM_022121	PERP, TP53 apoptosis effector
27*	213822_s_at	0.0133	UBE3B	BE856776	ubiquitin protein ligase E3B
28	204412_s_at	0.015	NEFH	NM_021076	neurofilament, heavy polypeptide 200 kDa
29	200862_at	0.0151	DHCR24	NM_014762	24-dehydrocholesterol reductase
30	210949_s_at	0.0153	EIF3S8	BC000533	eukaryotic translation initiation factor 3, subunit 8, 110 kDa
31	218393_s_at	0.0172	SMU1	NM_018225	homolog of C. elegans smu-1
32	221550_at	0.018	COX15	BC002382	COX15 homolog, cytochrome c oxidase assembly protein
33*	203413_at	0.0192	NELL2	NM_006159	NEL-like 2 (chicken)
34	221036_s_at	0.0192	PSFL	NM_031301	anterior pharynx defective 1B-like
35*	210915_x_at	0.0208	PRSS1	M15564	T-cell receptor precursor
36	218943_s_at	0.0212	DDX58	NM_014314	DEAD/H (Asp-Glu-Ala-Asp/His) box polypeptide
37	207813_s_at	0.0215	FDXR	NM_004110	ferredoxin reductase
38	221021_s_at	0.0216	CTNNBL1	NM_030877	catenin, beta like 1
39	214316_x_at	0.0216	MNT	AI378706	Calreticulin
40**	209728_at	0.022	HLA-DRB4	BC005312	major histocompatibility complex, class II, DR beta 3
41	215230_x_at	0.0229	EIF3S8	AA679705	eukaryotic translation initiation factor 3, subunit 8, 110 kDa
42	219269_at	0.024	FLJ21616	NM_024567	hypothetical protein FLJ21616
43**	207533_at	0.0246	CCL1	NM_002981	chemokine (C-C motif) ligand 1
44	207275_s_at	0.0255	ACSL1	NM_001995	acyl-CoA synthetase long-chain family member 1
45	217856_at	0.0274	RBM8A	AF182415	RNA binding motif protein 8A
46	200647_x_at	0.0277	EIF3S8	NM_003752	eukaryotic translation initiation factor 3, subunit 8, 110 kDa
47*	214395_x_at	0.028	PP3856	AI335509	eukaryotic translation elongation factor 1 delta
48**	211900_x_at	0.0297	CD6	U66146	CD6 antigen
49	215606_s_at	0.0307	ELKS	AB029004	Rab6-interacting protein 2
50	218945_at	0.0311	MGC2654	NM_024109	hypothetical protein MGC2654
51	209790_s_at	0.0314	CASP6	BC000305	caspase 6, apoptosis-related cysteine protease
52	201143_s_at	0.0325	EIF2S1	BC002513	eukaryotic translation initiation factor 2, subunit 1 alpha, 35 kDa
53	211965_at	0.0332	ZFP36L1	BE620915	zinc finger protein 36, C3H type-like 1
54**	201242_s_at	0.0339	ATP1B1	BC000006	ATPase, Na+/K+ transporting, beta 1 polypeptide
55	213388_at	0.0346	PDE4DIP	H15535	ym27c01.s1 Soares infant brain 1NIB cDNA clone IMAGE:49385 3'
56	214182_at	0.036	ARF6	AA243143	ADP-ribosylation factor 6
57	212190_at	0.0362	SERPINE2	AL541302	AL541302 Homo sapiens PLACENTA cDNA clone CS0DE006YI10 5'
58*	204015_s_at	0.0365	DUSP4	BC002671	dual specificity phosphatase 4
59**	206366_x_at	0.0372	XCL1	U23772	chemokine (C motif) ligand 1
60*	216609_at	0.0374	TXN	AF065241	Thioredoxin
61	219836_at	0.038	ZBED2	NM_024508	zinc finger, BED domain containing 2
62**	208602_x_at	0.0383	CD6	NM_006725	CD6 antigen
63**	208302_at	0.0385	HB-1	NM_021182	minor histocompatibility antigen HB-1
64	209433_s_at	0.0393	PPAT	AI457120	phosphoribosyl pyrophosphate amidotransferase
65**	221618_s_at	0.0404	TAF9L	AF220509	TAF9-like RNA polymerase II
66	202807_s_at	0.0427	TOM1	NM_005488	target of myb1 (chicken)
67	222145_at	0.043		AK027225	Similar to PI-3-kinase-related kinase SMG-1 isoform 1; (LOC390682)
68**	212599_at	0.0435	AUTS2	AK025298	autism susceptibility candidate 2
69	209392_at	0.0447	ENPP2	L35594	ectonucleotide pyrophosphatase/phosphodiesterase 2 (autotaxin)
70	204137_at	0.0453	TM7SF1	NM_003272	transmembrane 7 superfamily member 1 (upregulated in kidney)
71	209015_s_at	0.0455	DNAJB6	BC002446	DnaJ (Hsp40) homolog, subfamily B, member 6
72	209013_x_at	0.0482	TRIO	AF091395	triple functional domain (PTPRF interacting)
73	215920_s_at	0.0488	LOC283970	AC002544	
74	221069_s_at	0.0492	LOC51204	NM_016360	clone HQ0477 PRO0477p
75	214741_at	0.0494	ZNF131	AW968301	zinc finger protein 131 (clone pHZ-10)
76	207485_x_at	0.0495	BTN3A1	NM_007048	butyrophilin, subfamily 3, member A1
77	220937_s_at	0.0496	SIAT7D	NM_014403	sialyltransferase 7D

### Expression of the mouse orthologue of *SPOCK1 *is not altered in a RTT mouse model

**Figure 2:**
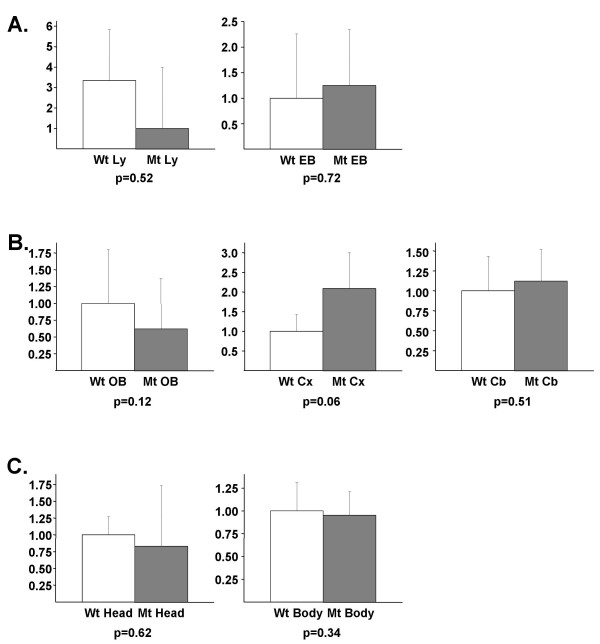
**Quantitative RT-PCR (qPCR) analysis of *Spock1 *expression**. Quantitative RT-PCR analysis of the expression of (**A**.) human *SPOCK1 *in the clonal lymphocyte RNAs (Mt Ly, Wt Ly) used for the microarray experiments and of mouse *Spock1 *in differentiating wild-type (Wt EB) and *Mecp2*^*R308/Y *^mutant (Mt EB) male mouse embryoid bodies; (**B**.) cerebral cortex (Wt Cx, Mt Cx), cerebellum (Wt Cb, Mt Cb) and olfactory bulb (Wt OB, Mt OB) of adult (5 week-old) wild-type and *Mecp2*^*R308/Y *^mutant male mice; and (**C**.) E16.5 embryo head (Wt head, Mt head) and body (Wt body, Mt body) (4 independent samples were tested for each tissue and genotype). The Y-axis shows the average fold change in expression +/- standard deviation) of mutant compared to wild-type, which is set as one-fold baseline, except for the Ly samples, where the one-fold baseline was set in the mutant for figure clarity (light bars = wild-type; dark bars = mutant), p-values are given under each respective graph.

Although not statistically significant, a trend for lower expression was found by qPCR analysis using the RNAs from the clonal lymphocyte cultures that were used to perform the microarray hybridizations, but no difference was seen in RNAs from differentiating wild-type and mutant mouse EBs (Figure 2A). To test if there is altered expression in the brain, the organ most affected by RTT-causing mutations, we performed qPCR reactions on RNA samples from mutant *Mecp2*^308/*Y *^and wild-type cerebral cortex, cerebellum and olfactory bulb from 5 week old mice (Figure 2B), as well as on heads and bodies from wild-type and mutant E16.5 embryos (Figure 2C). No statistically significant differences in expression of *Spock1 *between any of these mutant and wild type samples were detected.

## Discussion

Heterozygous mutations in X-linked *MECP2 *are found in most girls with RTT. The *MECP2 *gene is subject to XCI and classic RTT patients typically have random XCI patterns [55,56]. Hence, patients with RTT have a mosaic distribution of cells expressing the wild-type *MECP2 *and cells expressing the mutant *MECP2 *allele in all of their tissues. Analysis of gene expression profiles on tissues from deceased girls with RTT will therefore be compromised by unpredictable patterns of XCI. In addition, the presence of functional "wild-type" cells will also dampen small effects on the expression levels of MeCP2 target genes. Postmortem tissues are mostly from girls at later stages of the disorder and their gene expression profiles may reflect the presence of concurrent disease.

Because central nervous system neurons are the primary cell types where the RTT syndrome pathology occurs, a pure population of neurons with the mutant *MECP2 *on the active X chromosome, compared to one with the wild-type *MECP2 *on the active X chromosome from the same individual would constitute an ideal sample set for analysis of gene expression profiles in the presence of *MECP2 *mutations, but such samples are unavailable from human patients and alternative strategies are needed.

*MECP2 *is expressed, albeit at lower levels, in nearly all other tissues, including T-lymphocytes. Although T-lymphocytes are not the primary cell type where the RTT phenotype manifests, previous experiments have shown that lymphocytes with the mutant *MECP2 *on the active X chromosome are at a growth disadvantage compared to those with the wild-type *MECP2 *on the active X chromosome [37]. This suggested to us that at least a subset of the MeCP2 target genes are dysregulated in this cell type and pure T-lymphocyte clonal cell lines may be a valuable source of RNA for gene expression profiling in Rett syndrome.

We isolated single T-lymphocytes from blood samples of RTT patients and clonally expanded them in order to compare *MECP2 *mutant and wild type expression in the same patient. Since only one form of *MECP2 *would be expressed in each isolated clone, we expected that effects on gene expression profiles would be more pronounced. Furthermore, by comparing these clonal T-lymphocytes from the same patient, we could eliminate changes resulting from interindividual genetic differences. A similar approach was taken by Traynor et al., 2002, but instead of lymphocytes they used lymphoblastoid cell lines and fibroblast clones [29]. Lymphoblastoid cells have the disadvantage that they are immortalized which can affect gene expression patterns, while fibroblast cultures can gain epigenetic differences as they divide in culture. Although the T-lymphocytes used in our experiment were also cultured, the number of cell divisions was small and the cells were not transformed.

Because we did not study the primary cell type that is responsible for the RTT pathology, we strove to increase the likelihood that any observed differences in gene expression levels between mutant and wild-type *MECP2*-expressing clones were relevant to Rett syndrome. We used samples from patients with two different types of mutations (p.T158M and c.1308-1309delTC), combined the results from these samples to focus only on genes with a consistent pattern of increased or decreased levels of expression in all mutant clones compared to wild type. We next compared these results to those of our other independent microarray experiment on *Mecp2*-mutant mouse EBs and to those published in the literature from expression profiling [29-31,44] or large-scale ChIP experiments [34]. The limited overlap in identified genes with up or downregulated expression between all these different lists cannot be easily explained, but could have a number of different reasons. The tissues, or cell lines studied and specific mutations varied between the studies. Human as well as mouse samples were analyzed and they originated from different stages of the disease process. More likely reasons may be that overall expression differences between mutant and wild-type MeCP2 RNA samples are subtle but affect a large number of genes. An alternate hypothesis is that MeCP2 primarily regulates expression of a limited number of genes in very specific cells or in a transient manner. Such changes would be difficult to detect with the strategies used in any of these expression profiling studies.

One of the genes identified by our comparative analysis was *SPOCK1*. Its mouse homologue encodes Spock1/testican, a Ca(2+)-binding proteoglycan predominantly located in the extracellular matrix. Although its function is incompletely understood, Spock1/testican contains six functional domains, several of which have a protease-inhibitor function [57]. There is an N-terminal hydrophobic signal sequence for secretion of the protein in the extracellular matrix, a cysteine-rich region, a Kazal-like homology domain with a putative seine-protease inhibitor role, comprised in several regions of homology to SPARC/osteonectin, two calcium-binding EF hand motifs, and a C-terminal domain with homology to thyrotropin cysteine protease inhibitors (CVCW or thyorglobulin-like domain) that has been shown to inhibit the lysosomal cysteine protease cathepsin-L [58]. *Spock1 *is highly enriched in brain and is associated with the postsynaptic region of a subpopulation of pyramidal neurons in the CA3 area of the hippocampus [54]. It is also found in the choroid plexus and is highly enriched in the central nervous system (CNS) during neuronal proliferation and migration [52]. It affects neuronal attachment and neurite outgrowth likely through a modulation of the extracellular matrix [53]. Quantitative RT-PCR experiments on the clonal lymphocyte cultures showed only a trend for downregulation. Quantitative RT-PCR experiments on brain regions of *Mecp2 *mutant mice also did not show consistent changes in expression. This suggests that *SPOCK1 *is not a direct target of MeCP2 and its downregulation in the microarray experiment might have been a false positive finding.

## Conclusion

To find new putative MeCP2 targets, we performed expression profiling on clonal lymphocyte cultures from RTT patients with two different *MECP2 *mutations and compared the gene expression profiles to those of other screens for MeCP2 targets. No statistically significant differences in expression were found upon stringent analysis of the data. A putative downregulated MeCP2-target gene, *SPOCK1*, did not have altered expression in brain regions in a mouse model of Rett syndrome.

## Competing interests

The author(s) declare that they have no competing interests.

## Authors' contributions

KNT and JML prepared the lymphocyte cultures, verified their clonality and prepared RNA. DSK prepared samples for microarray analysis. IJD did the microarray data analysis, RT-PCR experiments and wrote the initial draft of the manuscript. JML and IBV were responsible for the concept, supervision and coordination of the experiments. IBV participated in data analysis and finalized the writing of the manuscript. All authors have contributed comments to the contents of the manuscript and have seen and approved the final submitted version.

## Pre-publication history

The pre-publication history for this paper can be accessed here:



## Supplementary Material

Additional File 1 Transformation of gene annotations to official human gene symbols for across-microarray comparisons. Click here for file

Additional File 2 Comparison of gene lists from different microarray experiments. Click here for file
