# Supplementary material for: Expression profiling of clonal lymphocyte cell cultures from Rett syndrome patients
Source: BMC Med Genet. 2006 Jul 21;7:61. doi: 10.1186/1471-2350-7-61 (PMC1569822; doi:10.1186/1471-2350-7-61)
Supplement: Additional File 2 — Comparison of gene lists from different microarray experiments. [file 1471-2350-7-61-S2.pdf]

SUPPLEMENTARY TABLE 2: COMPARISON OF GENE LISTS FROM DIFFERENT MICROARRAY EXPERIMENTS

Legend: gene symbols in parentheses indicate opposite regulation between the overlapping experiments.

	GCRMA lymphocytes	Traynor	Tudor	Colantuoni	Horike	Ballestar	Embryoid bodies
**GCRMA lymphocytes**		BEXL1 (SVIL)	none	(YWHAB)	none	none	LPIN, SPOCK1
**Traynor**	BEXL1 (SVIL)		none	none	none	none	CALD1, TCF21, C22orf16
**Tudor**	none	none		PVALB	none	none	none
**Colantuoni**	(YWHAB)	none	PVALB		none	none	CRYAB, C5orf13, CDC42
**Horike**	none	none	none	none		none	none
**Ballestar**	none	none	none	none	none		none
**Embryoid bodies**	LPIN, SPOCK1	CALD1, TCF21, C22orf16	none	CRYAB, C5orf13, CDC42	none	none	
